# Neural activity during response inhibition associated with improvement of dysphoric symptoms of PTSD after trauma-focused psychotherapy—an EEG-fMRI study

**DOI:** 10.1038/s41398-021-01340-8

**Published:** 2021-04-14

**Authors:** Richard A. Bryant, Thomas Williamson, May Erlinger, Kim L. Felmingham, Gin Malhi, Mark Hinton, Leanne Williams, Mayuresh S. Korgaonkar

**Affiliations:** 1 School of Psychology, University of New South Wales, Sydney, NSW Australia; 2 Brain Dynamics Centre, Westmead Institute for Medical Research, University of Sydney, Sydney, NSW Australia; 3 School of Psychological Sciences, University of Melbourne, Melbourne, VIC Australia; 4 Department of Psychiatry, University of Sydney, Sydney, NSW Australia; 5 Phoenix Australia, University of Melbourne, Melbourne, VIC Australia; 6 Department of Psychiatry and Behavioral Sciences, Stanford University, Stanford, CA USA; 7 Sierra-Pacific Mental Illness Research, Education, and Clinical Center (MIRECC) VA Palo Alto Health Care System, Palo Alto, CA USA; 8 School of Health Sciences, Faculty of Medicine and Health, University of Sydney, Sydney, NSW Australia

**Keywords:** Human behaviour, Prognostic markers

## Abstract

Although trauma-focused cognitive behavioural therapy (TF-CBT) is the frontline treatment for posttraumatic stress disorder (PTSD), up to one half of patients do not respond optimally to this treatment. Inhibitory functions are important for successful management of PTSD, yet there is a dearth of knowledge regarding the extent to which neural mechanisms unpinning response inhibition are associated with TF-CBT response. Treatment-seeking PTSD patients (*n* = 40) were assessed during a response inhibition task (the Go/No-Go task) while undergoing functional magnetic imaging (fMRI) and event-related potentials (ERP) in separate sessions. PTSD symptom severity was assessed with the Clinician-Administered PTSD Scale, before undergoing nine sessions of TF-CBT. They were then reassessed post-treatment to estimate reduction in fear and dysphoric symptoms of PTSD. Although neural responses during the inhibitory task did not predict overall symptom change, reduced activation in the left precuneus and the right superior parietal cortex predicted greater improvement in dysphoric symptoms. ERP responses during response inhibition indicated that lower P3 peak latency predicted greater reduction of dysphoric symptoms. There were no significant predictors of changes of fear symptoms. These findings indicate that neural activity associated with response inhibition can act as a predictive biomarker of TF-CBT response for PTSD symptoms. This pattern of findings underscores the importance of delineating the role of biomarkers to predict remission of subtypes of PTSD.

## Introduction

Trauma-focused cognitive behavior therapy (TF-CBT) is the primary frontline treatment for posttraumatic stress disorder (PTSD). Despite the demonstrated success of this treatment for many patients, 30–50% of those with PTSD do not respond to this therapy^1,2^. This situation has led to many attempts to understand predictors of treatment response, including neural markers that can predict who will respond to TF-CBT. The majority of these studies have focused on functional magnetic resonance imaging (fMRI) during emotional processing or regulation tasks^3–6^. Other studies have employed resting state MRI to identify neural profiles characteristic of TF-CBT responders^7^. These studies have led to variable findings that have not provided consistent regions that predict TF-CBT outcome; nonetheless, one review indicates that there is convergence that better treatment response is associated with increased pre-treatment dorsal anterior cingulate activation and decreased amygdala and insula activation^8^.

Inherent in most theories of TF-CBT is that successful treatment response involves the capacity to recruit inhibitory functions because one needs to manage trauma memories and strong negative emotional states^9^. There is convergent evidence that PTSD is associated with impaired executive functions^10^, and this includes deficits in inhibitory functions^11–14^. Deficits in inhibitory function are associated with severity of PTSD symptoms^15^, and may be particularly associated with level of re-experiencing symptoms^16^. Consistent with these findings, people with PTSD display less activation of the prefrontal cortex during inhibition tasks relative to controls^17–19^. There is evidence that the deficit in inhibitory functioning in PTSD may be associated with deficits in fear inhibition in that fear-potentiated startle and extinction to a conditioned stimulus are associated with reduced ventromedial PFC activation during a Go/No-Go paradigm^19^. The observation that fear inhibition may involve generic capacity of inhibitory functions in PTSD raises the possibility that propensity to activate inhibitory neural networks may be influential in how PTSD patients respond to TF-CBT. Several studies have investigated how response inhibition in PTSD patients may be associated with TF-CBT response. One small pilot study showed that reduced activation of frontal and left dorsal striatal networks during a Go/No-Go task predicted poorer response to TF-CBT^20^. Another study employed a response inhibition task that allowed delineation of contextual processing and inhibition, and found that treatment responders had increased activation of the inferior parietal lobe during contextual processing that non-responders^21^.

Reflecting temporal patterns during response inhibition, PTSD patients display aberrant evoked response potentials (ERPs) during Go/No-Go tasks. PTSD has been associated with longer latency of the P3 component^12,22^ and a shorter N2 latency^23^ during inhibition trials. One study also found that people with PTSD had larger P3 amplitude on both Go and No/Go trials^24^. Whereas the N2 is a negative component elicited approximately 200 ms after a Go/No-Go stimulus (potentially reflecting the early detection of the conflict between Go and No/Go), the P3 is a positive component elicited approximately 300 ms after the stimulus (and potentially represents a later stage of response inhibition involving decision processes^25,26^). Despite the evidence regarding distinct ERP patterns in PTSD patients during Go/No-Go tasks, this important temporal index of response inhibition has not been investigated in relation to predicting response to TF-CBT.

To date there have been no studies that have investigated the relationship between TF-CBT response and comprehensive assessment of inhibitory functions, including both fMRI and ERP measures. Using both indices is important because it allows assessment of both spatial and temporal measures of neural mechanisms of response inhibition. To this end, this study administered treatment-seeking PTSD patients (and healthy controls) a Go/No-Go task during separate fMRI and ERP recordings. PTSD patients then underwent a course of TF-CBT. On the basis that TF-CBT response involves intact inhibitory functions, we hypothesized that better treatment response would be predicted by stronger activation of prefrontal cortical networks and shorter P3 latencies during the Go/No-Go task.

We were also interested in how neural responses could predict remission of different subtypes of PTSD. Increasing evidence points to the heterogeneity of PTSD^27^, with evidence of two latent factors that comprise fear (including re-experiencing, active avoidance, hypervigilance, and elevated startle) and dysphoric (passive avoidance, sleep disturbance, concentration difficulties, and irritability) symptoms of PTSD^28,29^. These latent symptom constructs potentially represent a neurobiologically meaningful approach to understand capacity to predict symptom remission because evidence indicates that depression may contribute distinctly to executive function deficits relative to PTSD^30,31^. Moreover, there is evidence of different neural profiles in PTSD patients with and without comborbid depression^32–36^, which may be attributed in part to the greater reward deficits in depression^36^. Accordingly, it may be an over-simplification to relate neural process to a global reduction of PTSD symptoms, and it may be more meaningful to consider how response inhibition may predict treatment response on these two dimensions. On the basis of evidence that both depression and PTSD are associated with greater parietal network recruitment during response inhibition^17,37^, we predict that reduced parietal activation may be associated with greater reduction of dysphoric symptoms, but not necessarily of fear symptoms.

## Materials and methods

### Participants

**Fig. 1:**
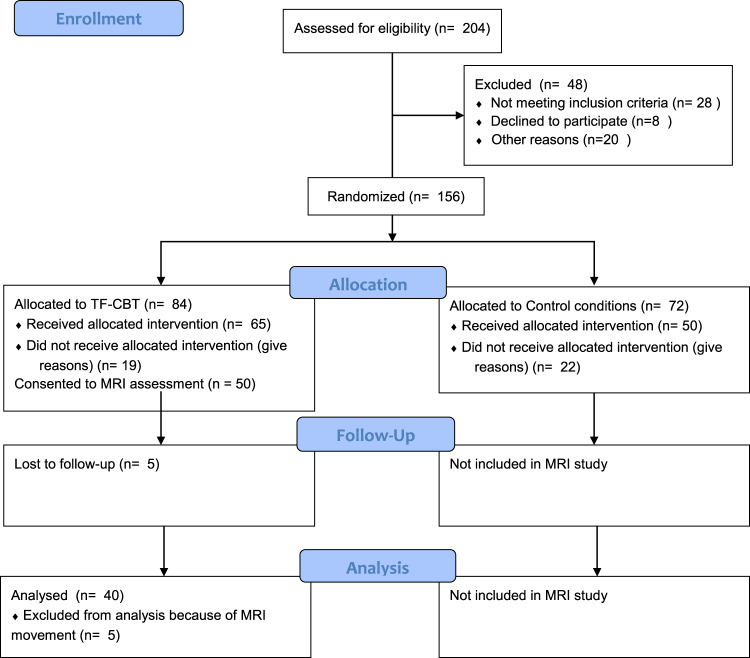
CONSORT flowchart of participant inclusion in the study.

Forty treatment-seeking PTSD patients (21 men, 19 women, mean age 40.6 years) participated in this study. Participants had experienced assault, childhood abuse, motor vehicle accidents, or police-related trauma leading to PTSD. Masters or doctoral-level clinical psychologists diagnosed DSM-IV PTSD used the Clinician-Administered PTSD Scale (CAPS^38^) to diagnose PTSD. The ‘2/1’ method was used to detect symptoms, indicating that a given symptom was experienced at least twice a month and caused moderate levels of distress. Participants were excluded if they reported substance dependence, bipolar disorder, psychosis, neurological disorders or moderate to severe brain injury (see Fig. 1 for Yesparticipant flow through the diagram). Medication was permitted as long as dosage had been stable for the previous two months and continued to do so for the course of the study. Selective serotonin reuptake inhibitors were used by 14 participants (35%). An age and gender matched healthy comparison group was also included, consisting of 40 participants (18 men, 22 women, mean age 39.4 years). Participants in this group had never experienced a Criterion A stressor and did not currently have an Axis I disorder, assessed using the Mini International Neuropsychiatric Interview (MINI version 5.5^39^). The Depression, Anxiety, and Stress Scale (DASS^40^) was used to measure levels of depression and anxiety in all patients. Table 1 details participant characteristics.

**Table 1:** Participant characteristics.

	PTSD	Controls	Responders	Non-responders
	(*n* = 40)	(*n* = 40)	(*n* = 27)	(*n* = 13)
Age, mean (SD)	40.6 (11.3)	39.4 (12.1)	41.5 (15.3)	38.8 (8.2)
Male, *n* (%)	21 (52.5)	18 (45)	13 (48.1)	8 (61.5)
Time since trauma, months, mean (SD)	16.7 (13.9)	—	18.4 (15.1)	13.3 (10.1)
Type of trauma, *n* (%)				
Childhood abuse	4 (10)	—	4 (14.8)	0 (0)
Motor vehicle accident	8 (20)	—	5 (18.5)	3 (23.1)
Police-related trauma	13 (32.5)	—	8 (29.6)	5 (38.5)
Assault	15 (37.5)	—	10 (37)	5 (38.5)
Prescribed SSRI, *n* (%)	16 (40)	—	11 (40.7)	5 (38.5)
Major depressive disorder, *n* (%)	15 (37.5)	—	9 (33.3)	6 (46.2)
Social phobia *n* (%)	1 (2.5)	—	0 (0)	1 (7.7)
Panic disorder, *n* (%)	7 (17.5)	—	5 (18.5)	2 (15.4)
Agoraphobia, *n* (%)	14 (35)	—	10 (37)	4 (30.8)
Generalised anxiety disorder, *n* (%)	8 (20)	—	6 (22.2)	2 (15.4)
Obsessive compulsive disorder, *n* (%)	2 (5)	—	2 (7.4)	0 (0)
Baseline CAPS severity, mean (SD)	71.8 (17.6)	—	72.6 (19.9)	70.2 (12.2)
Baseline Fear severity	39.9 (9.5)	—	39.9 (9.5)	39.9 (9.7)
Baseline dysphoria severity	31.3 (10.7	—	31.8 (12.3)	30.2 (6.2)
DASS Depression, mean (SD)	10.9 (5.2)	—	10.3 (5.5)	12.2 (4.4)
DASS anxiety mean (SD)*	8.1 (4.4)	—	7.3 (4.8)	9.2 (2.3)
Post-treatment CAPS severity, mean (SD)*	28.1 (20.0)	—	19.0 (14.5)	47 (16.5)
Post-treatment fear severity*	13.8 (11.3)	—	8.7 (7.6)	24.5 (10.5)
Post-treatment dysphoria severity*	14.1 (10.7)	—	10.1 (8.3)	22.2 (10.7)

Asterisks mark significant differences between treatment responders and non-responders (*p* < 0.05).

### Procedure

The Western Sydney Area Health Service Human Research Ethics Committee approved this study, and all participants gave written consent to partake in the study. Following assessment of PTSD using the CAPS, clinical psychologists used the MINI to assess for current major depressive episode (MDE), generalized anxiety disorder, social phobia, panic disorder, agoraphobia, obsessive compulsive disorder, and substance use disorder. Participants underwent clinical and lab (EEG and MRI) assessments at baseline, underwent a 9-week TF-CBT treatment course, and were assessed for PTSD severity following treatment.

### Go/No-Go task

The Go/No-Go task assesses response inhibition. Participants were instructed to respond to ‘Go’ trials by pressing a button as quickly as possible and withhold a response on ‘No-Go’ trials. The ‘Go’ stimuli was the word “PRESS” in green writing, and the ‘No-Go’ stimuli was the word “PRESS” in red writing. Each stimulus was presented for 500 ms, with a 750 ms interstimulus interval. In total, 180 Go stimuli and 60 No-Go stimuli were presented in a pseudorandom order to ensure that the No-Go stimulus did not occur more than three times in a row. Because the Go/No-Go task requires an all-or-nothing decision to either act or refrain from acting, it reflects participants’ abilities in inhibitory control. The Go/No-Go task was repeated three times in the session for each participant. We measured performance on the test, including commission errors (failing to withhold a response), omission errors (failing to correctly respond), and reaction time. The first task was conducted without neural recordings. The task was then repeated while continuous electroencephalogram (EEG) data was being recorded and subsequently during magnetic resonance imaging machine.

### Treatment protocol and analysis

Approximately 2 weeks after completing the Go/No-Go testing, participants began a 9-week TF-CBT treatment administered by experienced doctoral-level or masters-level clinical psychologist. Sessions occurred weekly and were 60–90 min in length. The therapy is consistent with prescribed TF-CBT protocols^41^. The TF-CBT comprised an initial session of psychoeducation about psychological responses to trauma, followed by six sessions of 40-min imaginal exposure to the trauma memory, implementation of in vivo exposure to avoided situations, and cognitive reframing of maladaptive thoughts related to the traumatic event. Two further sessions reinforced cognitive restructuring exercises, and a final session focused on relapse prevention^42^. This trial was prospectively registered at Australian and New Zealand Clinical Trials Registry, ACTRN12612000185864.

The change in PTSD symptom severity across treatment was calculated by subtracting post-treatment CAPS scores from pre-treatment scores. This score then was divided by the pre-treatment CAPS score, to produce a CAPS-change score independent of initial symptom severity. This change in PTSD severity score was used for correlation with task measures. To examine change in PTSD severity, while considering the heterogeneity of the disorder, change in CAPS factor scores (Fear factor scores and Dysphoria factor scores) were also calculated in the same way as the overall CAPS-change scores, and were correlated with neural measures. Fear factor was defined as total score of re-experiencing, active avoidance, hypervigilance, and elevated startle symptoms and dysphoric factor as a total score of passive avoidance, sleep disturbance, concentration difficulties, and irritability symptoms^29^.

### Behavioural/clinical data and analyses

Baseline clinical data (DASS Depression, Anxiety and Stress scores, type of trauma, time since trauma, comorbidities), and behavioral Go/No-Go data (commission errors, omission errors, reaction time) were tested for association with PTSD symptom improvement using Pearson correlations. Regressions analyses were conducted to examine the strength of each clinical or behavioural measure as a predictor of symptom improvement.

### EEG acquisition and analyses

Electrophysiological data was continuously recorded from 32 EEG channels at 500 Hz with a skin resistance of <5 kOhms, using a Quick Cap and NuAmps DC system (Neuroscan). Twenty six cephalic sites, 4 electro-oculogram (EOG) sites, an orbicularis oculus site, and a masseter site comprised the 32 channels. To record horizontal eye movement, electrodes were placed 1.5 cm lateral to the outer canthus of each eye. To record vertical eye movement, electrodes were placed 3 mm above the left eyebrow and 1.5 cm below the left lower eyelid. The threshold for artefact rejection was set at 100 µV. Each event-related potential epoch was filtered using a low-pass Tukey filter. No-Go trials were averaged together to form ERP waves, which were then hand-scored to determine the peak-value and latency of the N2 and P3 peaks. The peak-value and latency of the N2 wave (defined as 180–220 ms) was examined for the Fz, FCz, and Cz electrodes. In addition to these, the Pz electrode was also included for analysis of the P3 wave (defined as 250–550 ms). These waveforms and electrodes were selected based on previous studies in response inhibition^26,43^.

To examine whether pre-treatment EEG data was associated with a reduction in PTSD symptoms, we conducted repeated-measures ANOVAs using the set of electrodes as a within-subjects variable and CAPS-change values as a between-subjects covariate. A 3 × 1 repeated-measures ANOVA was conducted on both the N2-amplitude and N2-latency data. Similar analyses were conducted on the P3-amplitude and P3-latency data using a 4 × 1 repeated-measures ANOVA. Further posthoc correlations between individual electrodes and CAPS-change values were conducted on significant electrode sets.

### fMRI acquisition and analyses

Functional MRIs were conducted on a 3.0 T GE Signa HDx scanner and an eight-channel head coil, using an echo planar imaging protocol. We collected 120 T2*-weighted functional volumes in each task run, with three dummy scans collected prior to the sequence to ensure magnetisation had stabilised to a steady state. Each volume consisted of 40 axial slices parallel to the intercommissural line, with 3.5 mm thickness, 2.5 s TR, 27.5 TE, and 90° flip angle. The field of view was 24 × 24 cm^2^ and the matrix size was 64 × 64. We also acquired a T1-weighted anatomical image with 1 mm^3^ isotropic voxel resolution to normalise the fMRI data to standard space. This was produced using a 3D spoiled gradient echo sequence in the sagittal plane with the following parameters: TR = 8.3 ms, TE = 3.2 ms, flip angle=11°, TI = 500 ms, NEX = 1, ASSET = 1.5, S/I frequency direction, 256 × 256 matrix size, and 180 contiguous 1 mm slices.

Statistical Parametric Mapping (SPM8, Wellcome Department of Neurology, London) software was used to realign, normalise (into standardised MNI space), and smooth neuroimaging data. fMRI images were realigned and unwarped to the initial image for the task run to correct for participant motion within the scanner. To normalise data into stereotactic MNI space, we used the FMRIB linear registration tool to co-register the functional data to the T1 anatomical scan, as well as using the FMRIB nonlinear registration tool to normalise the weighted 3D spoiled gradient echo sequence. A mask covering the ventricles and white matter was used to estimate their corresponding signal and correct for any physiological noise. Smoothing was conducted on all fMRI data using an 8 mm Gaussian kernel. For first-level analysis, the BOLD response was estimated by convolving the hemodynamic response with a boxcar function in a general linear model framework. Contrast images for response inhibition were determined by comparing the No-Go versus the implicit baseline.

We examined brain areas making up the default mode network (DMN) and the cognitive control network (CCN) based on previous meta-analyses and studies, which have outlined the role of these networks in response inhibition processes^44,45^. These regions of interest (ROIs) were defined using 10 mm radius spheres combined into a single network specific mask. The regions of the DMN consisted of the medial prefrontal cortex (mPFC), the posterior cingulate cortex (PCC) and precuneus. The regions tested as part of the CCN were the dorsolateral prefrontal cortex (DLPFC), dorsal anterior cingulate cortex (dACC), inferior parietal, and superior parietal cortices. We also conducted exploratory analyses at the whole-brain level. All analyses were conducted voxel-wise and judged significant at a family-wise-error-corrected peak *p*-value of 0.05.

We first evaluated group differences between the PTSD and healthy control groups using a voxel-wise two-sample *t*-test on their respective contrast images. Next, the change in PTSD symptom severity score was regressed (voxel-wise) onto the pre-treatment fMRI signal to determine whether neuroimaging data was associated with reduction in PTSD symptoms; we also repeated analysis with reduction in fear and dysphoria symptoms. Finally, we conducted generalised psychophysiological interaction (gPPI) analyses^46^ to determine whether functional connectivity across DMN and CCN brain regions were associated with reductions in PTSD symptoms. Significant clusters from activation analyses were used as seeds to evaluate connectivity to the rest of the network regions. As done for the activation analyses, a family-wise error (FWE) corrected *p* level of 0.05 was used for statistical inferences.

### Additional analyses

We conducted a few additional analyses. First, we evaluated group comparisons between the PTSD and healthy control on the neurocognitive, EEG and fMRI measures, particularly to test if the identified prognostic measures also characterize PTSD diagnosis. Next, we performed analyses to build predictive models of treatment outcome using all data. For this using hierarchical stepwise linear regression models, we evaluated the additive predictive utility of each of our measures. Specifically, our goal was to determine whether the neural data (EEG and MRI) provided additional useful predictive information above that of the clinical and cognitive behavioral measures. For this we created a regression model in which the clinical and cognitive data were entered into a first block, and the neural measures (i.e. the significant EEG measures and fMRI clusters) were entered using a stepwise method in the second and third blocks, respectively. As an exploratory analysis to find the best overall predictive model, we used all the measures which were significantly correlated with change in CAPS (clinical, cognitive, EEG, and fMRI) using a stepwise regression and cross-validation analyses. For this analysis, we used a binary response variable based on 50% decrease in symptoms (i.e. responders and non-responders) to quantify treatment outcome. We used this analysis only to identify the most predictive features from the identified significant measures. We also ran cross-validation analyses on both these analyses to test generalizability of these models beyond our tested cohort. This linear cross-validation was conducted using bootstrapping in R. The data were randomly split into equal-sized training and test datasets (20 participants in each dataset). The training dataset was bootstrapped 200 times, with each bootstrap fitted with a linear regression. The averaged coefficients obtained from the bootstrapped training data were then applied to the test data to determine the generalizability of the model. To test whether our results were affected by SSRI use within the patient group, we repeated all analyses with a smaller group excluding participants who were currently taking SSRI medication.

## Results

### Clinical outcomes

All participants met the DSM-IV PTSD criteria and completed 7–9 sessions of TF-CBT. Mean CAPS scores prior to treatment was 71.8 ± 17.6, which reduced to 28.13 ± 20.0 at the post-treatment assessment. Twenty-seven (67.5%) participants were classified as treatment responders (50% or greater reduction in PTSD symptoms) and 13 (32.5%) were non-responders. Table 1 describes characteristics of the sample. We investigated whether clinical data collected prior to treatment was related to treatment outcome. Of the clinical measures, only DASS Anxiety scores significantly correlated with change in overall CAPS scores (*r* = −.409, *p* = .013), as well as change in dysphoria subscale scores (*r* = −.347, *p* = .038) and fear subscale scores (*r* = −.399, *p* = .016), with lower pre-treatment levels of anxiety predicting better treatment response.

### Go/No-Go performance

Behavioral data for the Go/No-Go task are detailed in the Supplementary Results (Table S1). These measures did not significantly correlate with change in overall CAPS scores or for fear or dysphoria symptoms. There were also no difference in Go/No-Go task performance between the PTSD and Control groups.

### EEG results

**Fig. 2:**
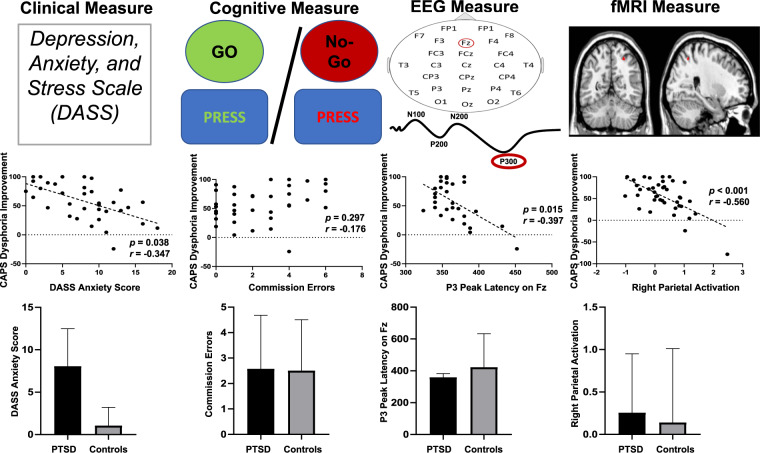
Associations between clinical and behavioural, EEG and fMRI measures of response inhibition with improvement in PTSD dysphoria symptoms. Pre-treatment measures of anxiety symptoms (DASS Anxiety), latency of P3 electrophysiology measurements and activation of right parietal and precuneus brain regions during response inhibition were associated with changes in PTSD dysphoria symptoms. The PTSD cohort was distinguished from controls only on pre-treatment DASS anxiety.

The latency of the P3 peak significantly correlated with change in CAPS dysphoria scores (*F* = 4.43, *p* = 0.045; shorter P3 latency associated with better improvement). Posthoc correlation analyses found that the P3 peak latency of the Fz electrode significantly correlated with CAPS Dysphoria change (*r* = −0.397, *p* = 0.03) (Fig. 2). This correlation did not survive correcting for the number of EEG measures tested (Bonferroni corrected *p* = 0.05/4 = 0.0125). However, when evaluated in the unmedicated PTSD cohort, this finding was significant at the Bonferroni corrected threshold (r = −0.459, *p* = 0.009, Supplementary Table S2). There were no significant correlations for the EEG measures with the change in overall CAPS or change in fear symptoms. There were also no differences in P3 peak latency of the Fz electrode between PTSD and control groups (see Table S3).

### fMRI activation

As observed with the EEG data, fMRI neural activation for the contrast reflecting response inhibition (No-Go vs Baseline) was significantly correlated with only change in dysphoria symptoms. Lower pre-treatment activation in the left precuneus of the DMN and right parietal cortex of the CCN were significantly associated with better improvement of dysphoric symptoms (see Table 2 and Fig. 1). However, when tested in the unmedicated PTSD sample, only the findings related to the right parietal cortex remained significant. There were no significant fMRI activation differences between the PTSD and control groups (Supplementary Table S4). Activation profiles for response inhibition for each group (Supplementary Tables S5 and S6) and exploratory whole-brain and ROI analyses for correlations with change in symptoms (Supplementary Table S7, S8, S9) are reported in supplementary section.

**Table 2:** fMRI neural activation predictive of PTSD symptom improvement for response inhibition.

Brain region	Direction	Peak MNI coordinates (X, Y, Z)	Cluster size in voxels	Peak z-score	*p*-value (FWE)
*Activity associated with total CAPS reduction*					
—	—	—	—	—	—
*Activity associated with reduction in fear symptoms*					
—	—	—	—	—	—
*Activity associated with reduction in dysphoria symptoms*					
L Precuneus	Negative	−4, −56, 46	110	3.79	0.014
R Parietal	Negative	30, −56, 52	14	3.73	0.027

### fMRI connectivity

We analyzed functional connectivity in a gPPI analysis with the left precuneus and right parietal regions as seeds. We found no connectivity with these regions, which correlated with changes in either the total CAPS or specific symptom scores. We also found no differences in connectivity between the PTSD and control groups for both seeds (Supplementary Table 10). Whole-brain connectivity profiles for both seeds for both PTSD and control group are also reported in supplementary section (Supplementary Tables S11 and S12).

### Predictive models

To test whether the neural data significantly improved the predictive model from using only the significant clinical measures (using the DASS Anxiety scores because this was the only clinical measure associated with symptom reduction), we performed a backward stepwise regression analysis using all the significant EEG and fMRI measures described above. The EEG indices associated with symptom change, specifically the latency of the P3 peak on the Fz electrode significantly improved the model relative to only using the DASS anxiety scores. Similarly, the fMRI activation in the right parietal cortex further improved the EEG-clinical model (see Table 3, Table S13).

**Table 3:** Predictive models using clinical, EEG and fMRI data.

Model	Predictors (β)	ANOVA		R²	R² Change	F change	Sig. F change
		F	Sig				
1	DASS anxiety (−2.89)	4.396	0.046	0.145	—	—	—
2	DASS anxiety (−2.77) Fz P3 latency (−0.61)	6.463	0.005	0.341	0.196	7.442	0.011
3	DASS anxiety (−2.60) Fz P3 latency (−0.52) Right parietal activity (−18.517)	6.345	0.003	0.442	0.101	4.367	0.047

There was a significant improvement in the clinical model after adding EEG measures and a further improvement after adding the fMRI measures.

To determine the best overall predictive model for reduction of PTSD dysphoria symptoms, we used a stepwise selection method with all significant predictors entered in a single block. The resultant model identified the same three measures as the best predictive features: DASS anxiety score, P3 peak latency on the Fz electrode, and activation in the right parietal cortex (Supplementary Table S14, Fig. S1).

## Discussion

This study aimed to evaluate the capacity of neural markers of inhibitory control to predict TF-CBT response by using a Go/No-Go paradigm during fMRI and ERP assessments. The major findings were that where there were no significant neural predictors of reduction of PTSD symptoms overall or fear symptoms, greater reductions in PTSD dysphoria symptoms were associated with (a) shorter P3 peak latency, and (b) lower activation in the left precuneus and right parietal cortex when participants inhibited response to No-Go stimuli. Further, the inclusion of these fMRI and ERP variables significantly improved prediction relative to clinical data alone; this model provided a cross-validated accuracy of 73% in predicting treatment response.

The finding that better treatment response was associated with shorter latency of the P3 component during inhibition needs to be understood in the context that this component represents a later phase of the inhibition process, possibly involving decision response^47^. Inhibitory performance has been shown to be deficient in PTSD^14^ and depression^48^, which is associated with longer latency of the P3 component^12,22^; these findings suggest that individuals with dysphoric symptoms of PTSD are less efficient in engaging inhibitory processes. The observation of shorter P3 latency predicting symptom remission suggests that those PTSD patients who have greater efficiency in inhibitory functions prior to treatment may be better able to utilize the strategies of TF-CBT and thereby achieve greater symptom remission.

Greater remission of dysphoria symptoms was associated with less activation in the parietal cortex and precuneus during response inhibition. Many studies have shown that a fronto-parietal network is strongly involved in response inhibition^49^. PTSD individuals demonstrate a reduced recruitment of this network during response inhibition^17^. Also, greater PTSD severity is found associated with greater parietal activation during this task^17^. There is also evidence of engagement of parietal regions during inhibition in patients with depressive states^37^. On the premise that the dysphoric phenotype of PTSD overlaps with depressive symptoms, it is possible that patients who do not experience remission of dysphoria symptoms may be deficient in inhibition processes and therefore engage inhibitory networks to a greater extent to compensate for this deficit. This interpretation accords with the finding of slower P3 predicting non-remission in more dysphoric patients because this pattern may reflect less efficient inhibitory processes.

It is interesting that both fMRI and ERP indices of response inhibition predicted remission of dysphoria, but not fear, symptoms of PTSD. This pattern underscores the heterogeneity of PTSD and points to the need for more nuanced and mechanistic approaches to identifying predictors of TF-CBT response instead of the prevailing focus on PTSD as a unitary construct. In this context it is worth noting that one large treatment study found that greater inferior parietal activation predicted remission of depression symptoms for SSRIs, but reduced activation predicted remission after serotonin and norepinephrine reuptake inhibitors (SNRIs)^50^. This pattern highlights that the role of inhibition in predicting treatment outcome appears to be specific to the treatment modality, and the current finding indicates that TF-CBT may implicate distinct inhibitory networks relative to antidepressants in predicting reduction of depressive symptoms of PTSD.

Although some models may suggest that response to TF-CBT should be associated with better inhibitory functions prior to treatment^51^, behavioral indicators of inhibition did not predict treatment response in the current study. This accords with other studies that have not reported an association between baseline neuropsychological measures of inhibition and treatment outcome^52^. Further, a meta-analysis of the relationship between neuropsychological measures of executive function found that response inhibition was not related to antidepressant response^53^. This pattern underscores the conclusion that biological markers may be predictive of treatment outcome even if behavioral performance is not.

In contrast to previous findings, the biological markers that predicted treatment outcome were not found to be distinct between PTSD and controls in our study. It is likely that the treatment response markers of specific PTSD symptoms could be different from those that characterize PTSD as an overall diagnosis. Interestingly, there were no EEG differences between PTSD and control participants. It should be noted that the body of evidence regarding distinct ERP patterns in PTSD during response inhibition is limited^12,22,23^, and the evidence points to no robust finding regarding any one ERP component’s latency or amplitude being distinctive to PTSD during this task. The current observation underscores the need for further study of EEG profiles in PTSD during response inhibition.

We recognize a number of limitations. First, this study lacked a no-treatment wait-list comparison condition, which would allow delineation between the predictive capacity of response inhibition for TF-CBT relative to spontaneous remission. Second, we only tested the predictive capacity of inhibition at post-treatment, and future studies should attempt to replicate this paradigm with longer-term follow-up assessments. Third, we note the modest sample size and acknowledge that a larger sample would allow for closer examination of PTD subtypes. Fourth, the predictive model yielded only modest accuracy, and so the current findings need to be replicated with larger and independent samples before any robust predictive model could be accepted.

In the context of precision psychiatry, there is currently much attention being given to the need for an evidence base that integrates neural indices that promotes matching of particular patients with specific treatments^54^. The current findings extend this goal by indicating that neural markers, such as those involving inhibitory processes, differentially predict remission of dysphoria, and fear symptoms of PTSD. Moreover, it shows that better prediction is achieved using multimodal assessments that integrate temporal and spatial indices of response inhibition. Future studies could usefully build on these findings by using complementary measures to assess different functions prior to treatment, and evaluate their predictive capacities for remission of distinct symptom phenotypes across different treatments. This approach is likely to yield more promising results than limiting the focus on diagnostic constructs.

## Supplementary information

Supplementary Results
