# Supplementary material for: Neural activity during response inhibition associated with improvement of dysphoric symptoms of PTSD after trauma-focused psychotherapy—an EEG-fMRI study
Source: Transl Psychiatry. 2021 Apr 14;11:218. doi: 10.1038/s41398-021-01340-8 (PMC8046805; doi:10.1038/s41398-021-01340-8)

**Supplementary Results**

Neural Activity During Response Inhibition Predicts Treatment Outcomes

in Posttraumatic Stress Disorder

**Table of Contents**

**Table S1.** Summary of behavioral data for the Go/No-Go task.

**Table S2.** Reanalysis of significant CAPS dysphoria correlations in the unmedicated portion of the sample.

**Table S3.** Summary of EEG analyses for response inhibition in a PTSD vs healthy control group comparison.

**Table S4.** Summary of voxel-wise whole brain activation analyses for response inhibition in a PTSD vs healthy control group comparison.

**Table S5.** Summary of one-sample voxel-wise whole brain activation analyses for response inhibition in PTSD group.

**Table S6.** Summary of one-sample voxel-wise whole brain activation analyses for response inhibition in healthy control group.

**Table S7.** Summary of voxel-wise whole brain activation analyses for response inhibition correlated with percentage change in PTSD symptom severity for NoGo-Baseline contrast.

**Table S8.** Summary of voxel-wise ROI activation analyses for response inhibition correlated with percentage change in PTSD symptom severity in regions activated in healthy controls and in the PTSD-HC comparison for NoGo-Baseline contrast.

**Table S9.** Summary of voxel-wise ROI activation analyses for response inhibition correlated with percentage change in PTSD symptom severity for NoGo-Go contrast.

**Table S10.** Summary of one-sample voxel-wise whole brain connectivity analyses for response inhibition in a PTSD vs healthy control group comparison.

**Table S11.** Summary of one-sample voxel-wise whole brain connectivity analyses for response inhibition in PTSD group.

**Table S12.** Summary of voxel-wise whole brain connectivity analyses for response inhibition in healthy control group.

**Table S13.** Cross-validated binary logistic regressions of clinical and neural measures of response inhibition using averaged coefficients from bootstrapped datasets to classify treatment responders and treatment nonresponders.

**Table S14.** Cross-validated linear regressions of clinical and neural measures of response inhibition using averaged coefficients from bootstrapped datasets to predict PTSD symptom improvement.

**Supplementary Results**

**Table S1.** Summary of behavioral data for the Go/No-Go task.

| **Behavioral measure** | **PTSD** | **Controls** | **Anova** | |
| --- | --- | --- | --- | --- |
|  | **Mean (SD)** | **Mean (SD)** | **F** | **Sig** |
| Commission Errors | 2.64 (2.08) | 2.53 (2.06) | 0.0038 | 0.846 |
| Omission Errors | 2.62 (4.25) | 3.82 (9.94) | 0.392 | 0.534 |
| Reaction Time | 352.73 (73.72 | 351.83 (104.92) | 0.001 | 0.971 |

**Table S2.** Correlation of significant clinical and neural markers to change in CAPS dysphoria scores in the unmedicated portion of the sample (N = 26)

We repeated correlation analyses between the changes in CAPS dysphoria and significant measures identified in the main text only on PTSD patients who were not taking SSRI medications. Of all the significant measures, the relationship between activation of left precuneus and change in CAPS dysphoria did not remain significant in unmedicated PTSD individuals.

| **Correlation with change in CAPS Dysphoria scores** | | |
| --- | --- | --- |
| **Variable** | ***r*** | ***p*** |
| *Clinical data* |  |  |
| DASS Anxiety | -0.365 | 0.036* |
| *EEG data* |  |  |
| Latency of P3 peak on Fz | -0.459 | 0.009* |
| *fMRI data* |  |  |
| Activation of left precuneus | -0.23 | 0.129 |
| Activation of right parietal cortex | -0.36 | 0.035* |

**Table S3.** Summary of differences between PTSD and controls for EEG measures of response inhibition. Repeated measures ANOVAs were conducted using the set of electrodes as a within-subjects variable compared across the PTSD and control groups. Posthoc t-tests were conducted on individual electrodes within significant electrode sets.

| **Electrode** | **Wave** | **Variable** | **F** | **t** | **p** |
| --- | --- | --- | --- | --- | --- |
| All | N200 | Amplitude | 6.507 | - | 0.014* |
| All | N200 | Latency | 0.501 | - | 0.483 |
| All | P300 | Amplitude | 0.903 | - | 0.347 |
| All | P300 | Latency | 1.739 | - | 0.194 |
| *Posthoc t-tests* | |  |  |  |  |
| Fz | N200 | Amplitude | - | 2.298 | 0.026* |
| FCz | N200 | Amplitude | - | 2.585 | 0.013* |
| Cz | N200 | Amplitude | - | 2.523 | 0.015* |

**Table S4.** Summary of voxel-wise whole brain & ROI activation analyses for response inhibition comparing PTSD and Controls. There were no significant clusters at *pFWE*<0.05 for the PTSD vs control group comparison hence findings at an uncorrected p<0.001 for whole brain analysis and uncorrected p<0.05 for ROI analysis are reported below.

| **Brain region** | **Direction** | **Peak MNI Coordinates (X, Y, Z)** | **Cluster size in voxels** | **Peak z-score** | | ***p*-value (uncorrected)** |  |
| --- | --- | --- | --- | --- | --- | --- | --- |
| *Whole Brain (p<0.001 uncorrected, >10 voxel cluster)* | | | | |  |  |  |
| R Inferior Parietal | Control > PTSD | 34, -46, 20 | 185 | 3.97 | | <0.001 |  |
| *Cognitive Control Network (p<0.05 uncorrected, >10 voxel cluster)* | | | | | | | |
| R Superior Parietal | PTSD > Control | 24, -66, 48 | 29 | 2.37 | | 0.009 |  |
| L Inferior Frontal | PTSD > Control | -38, 18, 24 | 21 | 1.93 | | 0.027 |  |
| *Default Mode Network (p<0.05 uncorrected, >10 voxel cluster)* | | | | | |  |  |
| n.s. | n.s. | n.s. | n.s. | n.s. | | n.s. |  |

**Table S5.** Summary of voxel-wise whole brain activation analyses for response inhibition. Significant clusters (*pFWE*<0.05) for one sample-tests for the PTSD group are reported below.

| **Brain region** | **Direction** | **Peak MNI Coordinates (X, Y, Z)** | **Cluster size in voxels** | **Peak z-score** | ***p*-value (FWE-corrected)** |
| --- | --- | --- | --- | --- | --- |
| *One sample t-test in PTSD patients* | | |  |  |  |
| L Supplementary Motor Area | Positive | -6, 4, 54 | 3358 | 7.78 | <0.001 |
| R Mid Cingulum | Positive | 6, 22, 34 |  | 5.96 | <0.001 |
| L Cerebellum | Positive | -34, -60, -28 | 6575 | 7.65 | <0.001 |
| L Cerebellum | Positive | -30, -76, -24 |  | 7.27 | <0.001 |
| R Cerebellum | Positive | 34, -58, -30 |  | 7.24 | <0.001 |
| L Insula | Positive | -42, 8, -4 | 820 | 6.12 | <0.001 |
| L Insula | Positive | -36, -8, -10 |  | 5.14 | <0.001 |
| L Postcentral Gyrus | Positive | -48, -10, 44 | 2177 | 6.11 | <0.001 |
| L Inferior Parietal | Positive | -40, -34, 34 |  | 6 | <0.001 |
| L Postcentral Gyrus | Positive | -52, -22, 46 |  | 5.69 | <0.001 |
| R Insula | Positive | 44, 18, -10 | 461 | 5.8 | <0.001 |
| R Insula | Positive | 46, 12, -2 |  | 5.79 | <0.001 |
| R Inferior Parietal | Positive | 42, -40, 32 | 980 | 5.46 | <0.001 |
| R Supramarginal | Positive | 54, -40, 42 |  | 5.24 | <0.001 |
| R Precentral Gyrus | Positive | 40, -20, 42 |  | 5.17 | <0.001 |
| L Supramarginal | Positive | -56, -22, 22 | 44 | 4.67 | <0.001 |
| L Cuneus | Negative | -14, -62, 24 | 580 | 5.71 | <0.001 |
| R Orbital MFG | Negative | 16, 44, -2 | 279 | 5.4 | <0.001 |
| R Orbital MFG | Negative | 8, 60, -8 |  | 5.25 | <0.001 |
| L Anterior Cingulum | Negative | -14, 48, -4 | 181 | 5.14 | <0.001 |
| L Orbital MFG | Negative | -8, 60, -8 |  | 5.03 | <0.001 |
| L Orbital SFG | Negative | -22, 42, -10 |  | 4.81 | <0.001 |
| R Precuneus | Negative | 20, -54, 20 | 70 | 5.08 | <0.001 |
| L Cuneus | Negative | 0, -80, 20 | 1 | 4.43 | <0.001 |
| L Anterior Cingulum | Negative | -6, 36, 0 | 1 | 4.41 | <0.001 |

**Table S6.** Summary of voxel-wise whole brain activation analyses for response inhibition. Significant clusters (*pFWE*<0.05) for one sample-tests for the control group are reported below.

| **Brain region** | **Direction** | **Peak MNI Coordinates (X, Y, Z)** | **Cluster size in voxels** | **Peak z-score** | ***p*-value (FWE-corrected)** |
| --- | --- | --- | --- | --- | --- |
| *One sample t-test in Healthy Controls* | |  |  |  |  |
| L Cerebellum | Positive | -36, -70, -26 | 7745 | Inf | <0.001 |
| L Cerebellum | Positive | -28, -80, -24 |  | Inf | <0.001 |
| R Cerebellum | Positive | 14, -84, -20 |  | 7.78 | <0.001 |
| L Supplementary Motor Area | Positive | -4, -4, 54 | 3139 | 7.52 | <0.001 |
| L Mid Cingulum | Positive | 0, -2, 32 |  | 5.47 | <0.001 |
| L Postcentral gyrus | Positive | -48, -10, 46 | 1673 | 7.48 | <0.001 |
| L Inferior Parietal | Positive | -46, -38, 36 |  | 5.69 | <0.001 |
| L Supramarginal | Positive | -48, -24, 34 |  | 5.39 | <0.001 |
| L Insula | Positive | -44, -10, -4 | 652 | 6.49 | <0.001 |
| L Rolandic Operculum | Positive | -54, -10, 0 |  | 5.13 | <0.001 |
| R Thalamus | Positive | 8, -16, 8 | 3456 | 6.25 | <0.001 |
| R Thalamus | Positive | 6, -22, 2 |  | 6.04 | <0.001 |
| R Midbrain | Positive | 12, -24, -16 |  | 5.99 | <0.001 |
| R Insula | Positive | 40, -4, -2 | 596 | 5.85 | <0.001 |
| R Superior Temporal | Positive | 50, -14, -6 |  | 5.72 | <0.001 |
| R Rolandic Operculum | Positive | 58, -8, 10 |  | 4.52 | <0.001 |
| R Inferior Parietal | Positive | 40, -40, 28 | 1834 | 5.79 | <0.001 |
| R Precentral Gyrus | Positive | 48, -4, 44 |  | 5.76 | <0.001 |
| R Supramarginal | Positive | 52, -40, 44 |  | 5.67 | <0.001 |
| R Middle Temporal | Positive | 50, -34, -8 | 346 | 5.57 | <0.001 |
| L Superior Temporal | Positive | -54, -46, 20 | 110 | 4.91 | <0.001 |
| L Cuneus | Negative | 2, -78, 24 | 862 | 6.1 | <0.001 |
| L Cuneus | Negative | -16, -56, 26 |  | 4.91 | <0.001 |
| R Cuneus | Negative | 14, -68, 20 |  | 4.87 | <0.001 |
| L Anterior Cingulum | Negative | -18, -44, -4 | 473 | 5.5 | <0.001 |
| R Anterior Cingulum | Negative | 20, -42, -4 |  | 5.43 | <0.001 |
| R Orbital MFG | Negative | 10, -44, -4 |  | 5.35 | <0.001 |

**Table S7.** Summary of voxel-wise whole brain activation analyses for response inhibition correlated with percentage change in PTSD symptom severity. Significant clusters (uncorrected *p*<0.001) for one sample-tests for the PTSD group are reported below.

| **Brain region** | **Direction** | **Peak MNI Coordinates (X, Y, Z)** | **Cluster size in voxels** | **Peak z-score** | ***p*-value (uncorrected)** |
| --- | --- | --- | --- | --- | --- |
| *Activity associated with total CAPS reduction* | | |  |  |  |
| R Caudate | Positive | 16, -12, 26 | 655 | 5.1 | <0.001 |
|  | Positive | -16, -14, 26 | 67 | 3.5 | <0.001 |
| R Orbital MFG | Negative | 4, 66, -4 | 31 | 3.83 | <0.001 |
| L Cingulum | Negative | -14, 22, 38 | 29 | 3.59 | <0.001 |
| L Calcarine | Negative | -18, -66, 12 | 35 | 3.57 | <0.001 |
| *Activity associated with reduction in fear symptoms* | | |  |  |  |
| R Caudate | Positive | 16, -12, 26 | 358 | 5.01 | <0.001 |
| *Activity associated with reduction in dysphoria symptoms* | | | |  |  |
| R Superior Parietal | Negative | 20, -72, 48 | 725 | 3.92 | <0.001 |
| R Precuneus | Negative | 14, -54, 44 |  | 3.92 | <0.001 |
| L Precuneus | Negative | -4, -54, 44 |  | 3.79 | <0.001 |
| L Parahippocampus | Negative | -22, -36, -10 | 132 | 3.85 | <0.001 |
| R Orbital MFG | Negative | 4, 64, -4 | 41 | 3.79 | <0.001 |
| L Inferior Parietal | Negative | -50, -32, 38 | 50 | 3.75 | <0.001 |
| L Calcarine | Negative | -16, -66, 14 | 70 | 3.7 | <0.001 |
| L Cuneus | Negative | -4, -80, 30 | 41 | 3.66 | <0.001 |
| L Superior Temporal | Negative | -48, 14, -4 | 35 | 3.59 | <0.001 |
| R Cuneus | Negative | 12, -70, 20 | 65 | 3.47 | <0.001 |
| R Superior Temporal | Negative | 60, -46, 12 | 41 | 3.43 | <0.001 |
| R Middle Temporal | Negative | 42, -68, 0 | 36 | 3.45 | <0.001 |

**Table S8.** Summary of voxel-wise ROI activation analyses for response inhibition correlated with percentage change in PTSD symptom for NoGo-Baseline contrast. ROIs were developed from regions active in healthy controls in a one-sample test and from regions differentially activated between PTSD and healthy controls. Clusters significant (*pFWE*<0.05) are reported below.

| **Brain region** | **Direction** | **Peak MNI Coordinates (X, Y, Z)** | | **Cluster size in voxels** | | **Peak z-score** | | ***p*-value (FWE-corrected)** | |  |
| --- | --- | --- | --- | --- | --- | --- | --- | --- | --- | --- |
| ***Activity associated with total CAPS reduction*** |  |  | |  | |  | |  | |  |
| *Healthy Control Activation Mask* | | |  | |  | |  | |  | |
| - | - | - | | - | | - | | - | |  |
| *PTSD vs Control Contrast Activation Mask* | | | | |  | |  | |  | |
| - | - | - | | - | | - | | - | |  |
| ***Activity associated with reduction in fear symptoms*** | | | | | | |  | |  | |
| *Healthy Control Activation Mask* | | |  | |  | |  | |  | |
| - | - | - | | - | | - | | - | |  |
| *PTSD vs Control Contrast Activation Mask* | | | | |  | |  | |  | |
| - | - | - | | - | | - | | - | |  |
| ***Activity associated with reduction in dysphoria symptoms*** | | | | | | |  | |  | |
| *Healthy Control Activation Mask* | | |  | |  | |  | |  | |
| - | - | - | | - | | - | | - | |  |
| *PTSD vs Control Contrast Activation Mask* | | | | |  | |  | |  | |
| R Caudate | Positive | 18 12 16 | | 17 | | 3.9 | | 0.025 | |  |
| R Caudate | Positive | 18 8 20 | | 1 | | 3.55 | | 0.045 | |  |

**Table S9.** Summary of voxel-wise ROI activation analyses for response inhibition correlated with percentage change in PTSD symptom severity for NoGo-Go contrast. There were no significant clusters at *pFWE*<0.05 hence findings at an uncorrected p<0.05 are reported below.

| **Brain region** | **Direction** | **Peak MNI Coordinates (X, Y, Z)** | **Cluster size in voxels** | **Peak z-score** | ***p*-value (uncorrected)** |
| --- | --- | --- | --- | --- | --- |
| ***Activity associated with total CAPS reduction*** |  |  |  |  |  |
| *Cognitive Control Network (P<0.05 uncorrected, >10 voxel cluster)* | | | | |  |
| R Mid Cingulum | Positive | 4 22 38 | 16 | 1.82 | 0.035 |
| *Default Mode Network (P<0.05 uncorrected, >10 voxel cluster)* | | | |  |  |
| R Angular | Positive | 46 -62 36 | 24 | 2.62 | 0.004 |
| ***Activity associated with reduction in fear symptoms*** | | |  |  |  |
| *Cognitive Control Network (P<0.05 uncorrected, >10 voxel cluster)* | | | | |  |
| R Mid Cingulum | Positive | 6 24 38 | 96 | 2.34 | 0.01 |
| *Default Mode Network (P<0.05 uncorrected, >10 voxel cluster)* | | | |  |  |
| R Angular | Positive | 46 -62 38 | 28 | 2.53 | 0.006 |
| ***Activity associated with reduction in dysphoria symptoms*** | | | |  |  |
| *Cognitive Control Network (P<0.05 uncorrected, >10 voxel cluster)* | | | | |  |
| R Mid Cingulum | Positive | 4 20 40 | 59 | 2 | 0.023 |

**Table S10.** Summary of voxel-wise whole brain & ROI connectivity analyses for response inhibition for unmedicated participants. There were no significant clusters (*p_FWE_*<0.05) for PTSD vs control group comparisons, hence clusters significant at an uncorrected p<0.001 for whole brain and uncorrected p<0.05 for ROIs are reported below.

| **Brain region** | **Direction** | **Peak MNI Coordinates (X, Y, Z)** | **Cluster size in voxels** | **Peak z-score** | ***p*-value (uncorrected)** |
| --- | --- | --- | --- | --- | --- |
| ***Connectivity with L Precuneus seed*** | | |  |  |  |
| *Whole brain (p<0.001 uncorrected, >10 voxel cluster* | | | |  |  |
| R Cerebellum | PTSD>HC | 20, -80, -22 | 89 | 4.28 | <0.001 |
| *Cognitive Control Network (p<0.05 uncorrected, >10 voxel cluster)* | | | | |  |
| R Middle Frontal | PTSD>HC | 36, 36, 34 | 156 | 2.56 | 0.005 |
| L Mid Cingulum | PTSD>HC | -4, 16, 34 | 33 | 2.01 | 0.022 |
| *Default Mode Network (p<0.05 uncorrected, >10 voxel cluster)* | | | | |  |
| L Angular | HC>PTSD | -42, -64, 30 | 52 | 2.27 | 0.012 |
| L Anterior Cingulum | HC>PTSD | -2, 42, 0 | 40 | 2.03 | 0.021 |
| L Posterior Cingulum | HC>PTSD | -2, -54, 32 | 20 | 1.96 | 0.025 |
| ***Connectivity with R Parietal seed*** | | |  |  |  |
| *Whole brain (p<0.001 uncorrected, >10 voxel cluster* | | | |  |  |
| R Mid Cingulum | HC>PTSD | 14, -16, 28 | 93 | 3.84 | <0.001 |
| *Cognitive Control Network (p<0.05 uncorrected, >10 voxel cluster)* | | | | |  |
| L Mid Occipital | PTSD>HC | -24, -66, 38 | 66 | 2.27 | 0.012 |
| *Default Mode Network (p<0.05 uncorrected, >10 voxel cluster)* | | | | |  |
| L Angular | HC>PTSD | -46, -64, 28 | 27 | 2.28 | 0.011 |
| L Precuneus | HC>PTSD | -12, -48, 46 | 47 | 2.27 | 0.011 |
| L Anterior Cingulum | HC>PTSD | -6, 48, 0 | 44 | 1.93 | 0.027 |

**Table S11.** Summary of voxel-wise whole brain connectivity analyses for response inhibition. Significant clusters (*p­_FWE_*<0.05) for one sample-tests for the PTSD group are reported below.

| **Brain region** | **Direction** | **Peak MNI Coordinates (X, Y, Z)** | **Cluster size in voxels** | **Peak z-score** | ***p*-value (FWE)** |
| --- | --- | --- | --- | --- | --- |
| ***One-sample t-test in PTSD*** | |  |  |  |  |
| *Connectivity with L Precuneus seed* | |  |  |  |  |
| L Precuneus | Positive | -4, -52, 44 | 7972 | Inf | <0.001 |
| R Middle Temporal | Positive | 50, -44, 6 | 1861 | 6.74 | <0.001 |
| L Thalamus | Positive | -6, -22, 2 | 214 | 5.8 | <0.001 |
| L Mid Cingulum | Positive | -4, 16, 32 | 826 | 5.72 | <0.001 |
| R Cerebellum | Positive | 20, -80, -22 | 94 | 5.34 | 0.001 |
| L Superior Temporal | Positive | -54, -38, 22 | 77 | 5.05 | 0.004 |
| L Inferior Frontal | Positive | -44, 38, 14 | 79 | 5.04 | 0.004 |
| R Thalamus | Positive | 10, -20, 0 | 124 | 4.97 | 0.005 |
| L Middle Frontal | Positive | -32, 34, 34 | 54 | 4.86 | 0.009 |
| L Supplementary Motor Area | Positive | -4, -4, 56 | 84 | 4.86 | 0.009 |
| R Postcentral Gyrus | Positive | 58, -8, 34 | 29 | 4.85 | 0.009 |
| *Connectivity with R Parietal seed* | |  |  |  |  |
| R Inferior Parietal | Positive | 28, -56, 50 | 13630 | Inf | <0.001 |
| R Midbrain | Positive | 12 -20, -4 | 490 | 5.92 | <0.001 |
| L Thalamus | Positive | -16, -22, 0 | 408 | 5.59 | <0.001 |
| L Middle Frontal | Positive | -24, 0, 54 | 67 | 5.28 | 0.001 |
| L Precentral | Positive | -46, 4, 18 | 88 | 5.24 | 0.002 |
| L Postcentral | Positive | -48, -10, 38 | 97 | 5.01 | 0.004 |

**Table S12.** Summary of voxel-wise whole brain connectivity analyses for response inhibition. Significant clusters (*p_FWE_*<0.05) for one sample-tests for the control group are reported below.

| **Brain region** | **Direction** | **Peak MNI Coordinates (X, Y, Z)** | **Cluster size in voxels** | **Peak z-score** | ***p*-value (FWE)** |
| --- | --- | --- | --- | --- | --- |
| ***One-sample t-test in Healthy Controls*** | |  |  |  |  |
| *Connectivity with L Precuneus seed* | |  |  |  |  |
| L Precuneus | Positive | -2, -56, 44 | 9645 | Inf | <0.001 |
| R Middle Temporal | Positive | 52, -48, 8 | 469 | 6 | <0.001 |
| L Middle Temporal | Positive | -48, -62, 6 | 217 | 5.56 | 0.001 |
| L Middle Temporal | Positive | -56, -36, -12 | 172 | 5.55 | 0.001 |
| L Superior Temporal | Positive | -54, -34, 18 | 133 | 5.51 | 0.001 |
| L Middle Frontal | Positive | -26, 22, 40 | 38 | 5.5 | 0.001 |
| L Cerebellum | Positive | -46, -54, -28 | 69 | 5.11 | 0.007 |
| L Middle Frontal | Positive | -44, 14, 38 | 30 | 4.94 | 0.015 |
| R Middle Frontal | Positive | 48, 10, 40 | 28 | 4.92 | 0.016 |
| R Calcarine | Positive | 16, -60, 18 | 38 | 4.88 | 0.02 |
| R Cingulate | Positive | 6, -6, 30 | 20 | 4.81 | 0.027 |
| *Connectivity with R Parietal seed* | |  |  |  |  |
| R Angular | Positive | 28, -58, 52 | 19244 | Inf | <0.001 |
| L Precentral | Positive | -56, 0, 28 | 403 | 6.13 | <0.001 |
| L Cerebellum | Positive | -34, -46, -34 | 1091 | 6.1 | <0.001 |
| L Inferior Occipital | Positive | -44, -68, -4 | 229 | 5.66 | <0.001 |
| R Middle Frontal | Positive | 34, 40, 28 | 216 | 5.64 | 0.001 |
| R Cerebellum | Positive | 34, -48, -34 | 325 | 5.36 | 0.002 |
| R Hippocampus | Positive | 28, -20, -10 | 128 | 5.08 | 0.008 |
| R Cerebellum | Positive | 22, -74, -18 | 41 | 4.98 | 0.012 |

**Estimation of prediction accuracy and cross validation analyses of neural measures to predict response to treatment.**

We ran linear and binary logistic cross-validation regression analyses to determine the prediction validity of our significant clinical and neural measures in predicting change in symptom from pretreatment to posttreatment, and to classify PTSD patients based on treatment outcome (responder and non-responder groups were characterized using 50% reduction in symptoms as threshold).

**Table S13.** Cross-validated binary logistic regressions using averaged coefficients from bootstrapped datasets for response inhibition. The table lists the overall accuracy, confidence interval, specificity, sensitivity, and the coefficient beta values of the cross-validated model, to determine which variable most contributes to the test model.

| ` | **Variable** | **Coefficent β values** | **% accuracy (95% CI)** | **% specificity** | **% sensitivity** | **Positive/Negative Predictive Value** |
| --- | --- | --- | --- | --- | --- | --- |
| *Cross-validated model* |  |  | 73.3% (45%, 92%) | 88.90% | 50% | 75.0%/72.7% |
|  | DASS Anxiety | -0.2 |  |  |  |  |
|  | Fz Latency on P3 Wave | -0.0351 |  |  |  |  |
|  | R Parietal | -1.59 |  |  |  |  |

**Table S14.** Cross-validated linear regressions using averaged coefficients from bootstrapped datasets for response inhibition. Extracted betas for the relevant neural and clinical measures are included in the linear regression model. The table lists the overall goodness of fit of the training and cross-validated models, and the coefficient beta values of the cross-validated model, to determine which variable most contributes to the test model.

|  | **Variable** | **Coefficient β values** | **Overall model *r²*** |
| --- | --- | --- | --- |
| *Training model* |  |  | 44.20% |
| *Cross-validated model* |  |  | 14.40% |
|  | DASS Anxiety | -2.323 |  |
|  | Fz Latency on P3 Wave | -0.619 |  |
|  | R Parietal | -20.709 |  |

**Figure S1. Receiver operating characteristic curve for the best model predicting change in CAPS dysphoria scores.** The best predictive model included a clinical measure (DASS Anxiety scores), an electrophysiological measure (P3 peak latency on the Fz electrode), and a functional imaging measure (activity in the right parietal cortex).


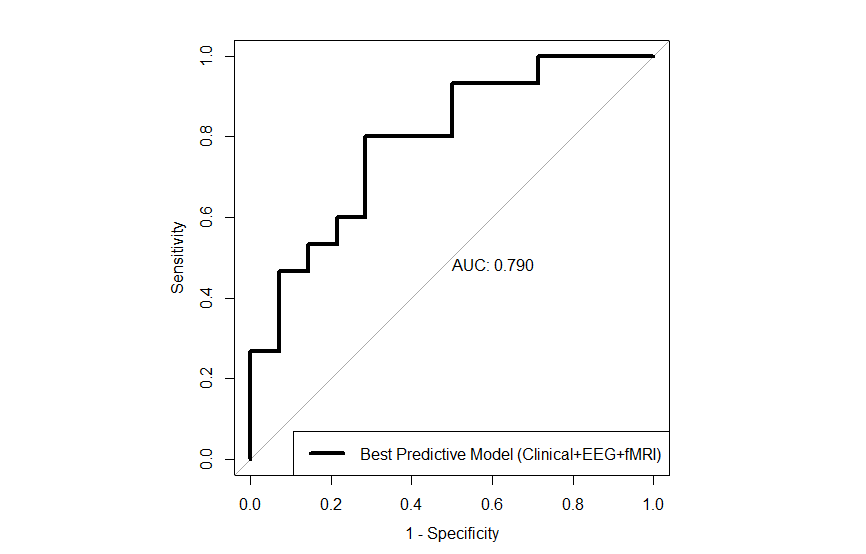

Supplement: Supplementary file 1 — Supplementary Results [file 41398_2021_1340_MOESM1_ESM.docx]
